# Changes in Respiratory Viruses’ Activity in Children During the COVID-19 Pandemic: A Systematic Review

**DOI:** 10.3390/jcm14041387

**Published:** 2025-02-19

**Authors:** Marco Maglione, Vincenzo Tipo, Emiliano Barbieri, Roberta Ragucci, Agnese Sara Ciccarelli, Chiara Esposito, Ludovica Carangelo, Antonietta Giannattasio

**Affiliations:** Pediatric Emergency Unit, Santobono-Pausilipon Children’s Hospital, 80129 Naples, Italy; v.tipo@santobonopausilipon.it (V.T.); emiliano.barbieri@unina.it (E.B.); ro.ragucci@unina.it (R.R.); agnesesara.ciccarelli@studenti.unicampania.it (A.S.C.); chiara.esposito@unina.it (C.E.); l.carangelo@santobonopausilipon.it (L.C.); a.giannattasio@santobonopausilipon.it (A.G.)

**Keywords:** children, COVID-19, acute respiratory infections, epidemiology, lockdown

## Abstract

**Background/Objectives**: The impact of the coronavirus disease 2019 (COVID-19) pandemic on health was significant worldwide. The measures adopted to limit the spread of the virus had an indirect effect on the epidemiology of other infectious diseases with similar mechanisms of inter-human transmission. The present literature review analyzed the scenario of pediatric acute respiratory infections in the post-lockdown period compared to the pre-pandemic and lockdown periods. The different patterns of viruses’ outbreaks were evaluated according to the type of local COVID-19 restrictive measures and to the type of pathogen. **Methods**: Relevant scientific literature published between March 2020 and November 2024 was identified by means of electronic keyword searches in the PubMed, Scopus, and Cochrane Library databases. **Results**: Worldwide implementation of non-pharmacological public health interventions aimed at limiting the COVID-19 pandemic resulted in a measurable effect on the circulation of other common respiratory viruses, significantly affecting their usual seasonality. Most viruses significantly reduced their activity during the lockdown period but returned to or exceeded historical levels after discontinuation of preventive non-pharmacological measures. For many respiratory viruses, particularly respiratory syncytial virus, an off-season increase was reported. **Conclusions**: The non-pharmacological interventions, which effectively helped limit the COVID-19 pandemic, resulted in relevant epidemiologic changes in most common respiratory viruses. Given the different seasonality and clinical severity observed for some pathogens after lockdown, possible future off-season or more severe epidemics should be expected.

## 1. Introduction

Acute respiratory infections (ARIs) are the leading infectious diseases among children worldwide and are significantly associated with pediatric hospitalization and death [1,2,3,4]. The application of multiplex reverse transcriptase polymerase chain reaction (RT-PCR) platforms to the analysis of sputum and deep throat swabs has allowed a better characterization of the etiology of viral ARIs. This kind of technique provides molecular detection of several common respiratory viruses and atypical bacterial pathogens with a turnaround time of few hours [5,6]. The identification of viral etiology allows a better understanding of the clinical features of respiratory tract infections and represents a valuable tool for epidemiological surveillance. Even though its impact on therapeutic strategies may appear limited, an early detection of viral pathogens in children with respiratory symptoms could avoid unnecessary antibiotic therapy, decrease the length of hospitalization, and reduce mortality, particularly from multiple infections [7]. Human rhinovirus/enterovirus (HRV), followed by adenovirus (ADV), has been reported as the commonest viral pathogen detected by PCR techniques in hospitalized children [7]. On the other hand, the significant disease burden and impact of respiratory syncytial virus (RSV) at a hospital level is well known [8].

The coronavirus disease 2019 (COVID-19) epidemic had an enormous impact on health worldwide since 2019. Soon after the outbreak, the epidemiology of infectious diseases dramatically changed. During the first pandemic year (pre-vaccine year), preventive interventions were mostly non-pharmacological (social distancing, mask-wearing, shelter-in-place, travel restrictions, school closure, etc.) [9,10]. Nevertheless, in addition to their undeniable impact on severe acute respiratory syndrome coronavirus 2 (SARS-CoV-2) diffusion, these measures resulted in preventing the spread of several other respiratory pathogens that are transmitted by large droplets from infected to susceptible people [11]. A review including studies up to 2019 analyzed the use of surgical masks during the common peak seasons of respiratory infections [9]. Unexpectedly, the authors observed that mask wearing was ineffective at limiting the spread of influenza [9]. Nevertheless, this finding may be explained by poor study design, insufficiently powered studies arising from low viral circulation in some studies, poor compliance with mask wearing, the quality of the masks used, self-contamination of the mask by hands, and a lack of protection from eye exposure to respiratory droplets [9]. Hand hygiene measures seemed to have a more important protective effect on the spread of respiratory viruses [9].

Routine long-term implementation of most non-pharmacological interventions may be problematic outside hospital settings and in non-pandemic periods. More effectively, the strict measures adopted during the COVID-19 pandemic would be applied in the event of future epidemics from other respiratory viruses. Indeed, large epidemics from respiratory viruses are likely to emerge, probably due to the re-circulation of viruses within immunologically naïve populations of infants born from mothers who have not reinforced their immunity [12].

The aim of this review is to describe the scenario of ARIs in the post-lockdown period compared to the pre-COVID-19 and lockdown periods, and to analyze the different patterns of outbreaks of the most relevant respiratory viruses in children according to the type of local COVID-19 restrictive measures.

## 2. Materials and Methods

### 2.1. Search Strategy

We carried out an electronic keyword literature search for English articles published on the epidemiology and clinical spectrum of pediatric ARIs from the beginning of the COVID-19 pandemic (March 2020) up to November 2024. The search strategy followed the Preferred Reporting Items for Systematic Reviews and Meta-Analysis (PRISMA) guidelines [13]. The PubMed, Cochrane Library, and Scopus databases were searched for articles with full-text availability using the following query: “respiratory tract infection OR influenza OR respiratory syncytial virus OR rhinovirus OR parainfluenza OR human metapneumovirus OR adenovirus OR bocavirus OR coronavirus OR SARS-CoV-2 AND children”. When available, the filters “humans” and “child: birth-18 years” were applied. The article search and data extraction were carried out in January 2025. The study protocol was not recorded in an official register.

### 2.2. Study Selection

All duplicates were removed through manual screening. Reference lists from the retrieved articles were manually searched to identify further eligible studies. Four independent reviewers (M.M., E.B., R.R., L.C.) screened the titles and abstracts, and eligibility was assessed in consensus in the event of disagreement among them. The full text of the selected studies underwent a further screening.

### 2.3. Inclusion and Exclusion Criteria

The inclusion criteria were as follows: (1) studies addressing epidemiology of one or more respiratory viruses in children during and/or after the COVID-19 pandemic; (2) the focus of the research was the impact of the COVID-19 pandemic on pediatric respiratory infections or the changes observed in the epidemiology of respiratory viruses throughout the pandemic years. The exclusion criteria were as follows: (1) irrelevance to the research topic; (2) articles addressing respiratory infections only before the COVID-19 pandemic; (3) articles focusing on specific populations (e.g., oncologic patients, neurologically impaired children); (4) articles mainly focusing on the genomic aspects of respiratory pathogens; (5) unavailability of free full-text.

### 2.4. Data Extraction

To confirm the relevance of each included study, the authors extracted the following data, which were reported in a data collection form: name of the first author, title, country, year of publication, study design.

### 2.5. Risk of Bias Assessment

The Newcastle–Ottawa Quality Assessment Scale [14] was used for assessing the risk of bias for each study. Three reviewers (M.M., E.B, A.G.) independently performed the evaluation. According to the assigned score, the studies were classified as low (<5 points), moderate (5–7 points), and high (>7 points) quality.

### 2.6. Outcomes

The analysis of the included studies considered the prevalence of various respiratory viruses in children during and after the COVID-19 pandemic as the main outcome. The change in this prevalence in relationship with the different phases of the pandemic represented the focus of the review.

## 3. Results

**Figure 1:**
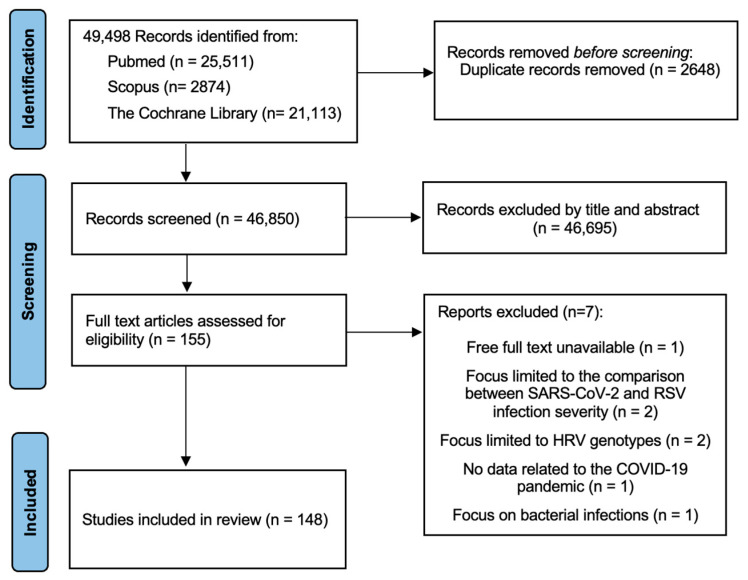
PRISMA flowchart for selection of studies [13].

After screening the databases, a total of 49,498 articles were returned. Of these, 155 articles were assessed for eligibility, and, after the full text evaluation, 148 were ultimately included in the review (Figure 1). A complete list of the included articles, with the first author’s name, country, and study design is reported in Supplementary Table S1. Supplementary Table S2 includes seven articles initially assessed for eligibility but ultimately excluded from the review after full text evaluation.

### 3.1. Characteristics of Included Studies

Most included studies had a retrospective observational design, with a minority of prospective observational studies (21/148), nine reviews and two commentaries. Approximately half of the included studies (71/137) were single-centered, whereas 66 were performed in multiple centers. The studies were conducted in 41 different countries. Quality was rated as high for 28 studies, and 56 were graded as moderate (Supplementary Table S1).

### 3.2. ARIs During the First Lockdown

At the beginning of COVID-19 pandemic, the adopted measures implemented to contain the spread of SARS-CoV-2 infection included stay-at-home-orders, school and business closures, travel restrictions, border closures and mandatory face coverings. These measures also affected the circulation of other respiratory viruses in almost all countries [10,15,16,17,18,19,20,21,22,23]. Not only the spread, but also the seasonality of most respiratory pathogens was altered during the first half of 2020 [16].

#### 3.2.1. Respiratory Syncytial Virus

RSV is the most common cause of acute bronchiolitis that may have a severe clinical presentation, particularly in high-risk patients, such as preterm infants or children with comorbidities [24,25]. RSV causes epidemics during the fall and winter seasons in temperate countries, as well as during the hot rainy seasons in tropical climates [24].

The suppression of RSV activity during the COVID-19 pandemic has been reported in almost all countries [11,25,26,27,28,29,30,31,32,33,34,35,36,37,38,39,40]. The European Centre for Disease Prevention and Control registered a fall in the detection of RSV from 2000 to 2500 cases per week at peak incidence during the previous four seasonal outbreaks (2016–2017, 2017–2018, 2018–2019, 2019–2020) to less than 700 cases per week in 2020–2021 [41]. The reduction in bronchiolitis led to a substantial reduction in pediatric emergency department visits [30] and in pediatric intensive care unit (PICU) admissions for severe cases [30,42,43].

In a study involving seven US pediatric medical centers, RSV detections in 2020 stopped after week 15 [26]. In 2020 no RSV cases were detected in weeks 15–18 (between 5 and 30 April) in either the emergency department or inpatient settings. The decrease in RSV-positive ARIs in the community mitigation period in 2020 compared to the same period in prior surveillance seasons was greater than 70% [26]. Although a multitude of papers on the epidemiology of RSV during COVID-19 are available, very few studies analyzed the clinical spectrum of rare cases of RSV infection during the lockdown [44].

#### 3.2.2. Influenza

Since February 2019, no seasonal influenza epidemic peak has been observed. In Australia, the reduction in influenza virus (IFV) detections in 2020 compared to 2017–2019 was 90% and 93% in children aged 0–4 and 5–14 years, respectively [45]. Another Australian study from Melbourne reported a 77.3% reduction in the proportion of positive IFV-A detections and an 89.4% reduction in the proportion of positive IFV-B detections during the COVID-19 restriction period compared to pre-pandemic data [46]. There were no IFV-A detections from week 14, 2020, and no IFV-B detections from week 17, 2020—2 weeks after the beginning of the initial lockdown. Similarly, levels of IFV transmission were very low in 2020 in a number of countries all over the world [40,47,48,49,50,51]; this was also true in China, where peaks of IFV-A infection appeared in February 2018 and 2019; the 2020 peak of IFV-A infection was reported in December [52].

A study from Korea analyzed the impact of social distancing on influenza activity in the community, collecting data on clinical influenza-like syndrome, laboratory parameters, and hospitalized cases. The authors reported substantially lower overall influenza activity in 2019–2020 compared to recent influenza seasons (2016–2017 and 2018–2019). The epidemic season terminated 8 to 12 weeks earlier, leading to a decrease in the influenza epidemic duration by 6–12 weeks. The influenza activity peak was lower, with 49.8 influenza-like illnesses/1000 visits compared to 71.9–86.2/1000 visits in previous seasons [53].

#### 3.2.3. Other Viral Infections

Most studies from several geographic areas reported a relevant decrease in the detection of respiratory viruses in children with symptoms of ARIs during the pandemic [54,55,56,57,58,59]. A study from Japan reported a drastic reduction in human metapneumovirus (HMPV) and Mycoplasma pneumoniae during the school closure period. In addition, almost no patients with these infections were hospitalized during the same period [54].

In a large study from China investigating the presence of eight viral pathogens (IFV, RSV, human parainfluenza virus, ADV, human coronavirus, human bocavirus, and HRV) in 314 sentinel hospitals, the impact of non-pharmacological interventions was evaluated in three subsequent periods based on the timeline of major interventions for containing the COVID-19 epidemic in China: 23 January to 7 April 2020 (Phase I), when the city of Wuhan was placed under lockdown, 8 April to 31 August 2020 (Phase II), when nationwide non-pharmacological interventions were relaxed but schools remained closed, and 1 September 2020 to 22 January 2021 (Phase III), when schools were re-opened in most provinces [16]. Overall, they observed the largest drop in the annual cumulative positive rate for IFV, with a reduction of 87.6%, followed by 70.6% for HMPV, 47% for ADV, and 32.3% for the parainfluenza virus [16]. Positive rates of almost all viruses rose significantly above historical levels during Phase III.

Interestingly, reports from different geographic settings have highlighted that, unlike most respiratory viruses, the drop in HRV prevalence was less marked when compared to other pathogens [60,61,62,63,64,65,66,67]. Indeed, it has been hypothesized that the social distancing restrictions during the lockdown were more effective at suppressing the other respiratory viruses than HRV, whose transmission was less altered [68]. Findings of this nature were further confirmed on a global scale by a large meta-analysis identifying HRV and RSV as the most prevalent among all respiratory viruses in children aged 0–12 years during the pandemic [69].

Likewise, HRV (human bocavirus) is reported to have been a significantly prevalent agent in pediatric ARIs during the pandemic period, often detected in association with other co-infecting pathogens, mainly RSV and HRV [70]. Similarly, although the positivity rate of ADV after January 2020 was lower than in the previous two years [52], this pathogen was detected to some extent during the pandemic, in contrast to the consistent reduction in other viruses [48,64,65,67].

These data suggest that standard prophylaxis against SARS-CoV-2 has limited effects in eliminating non-enveloped viruses [64].

### 3.3. ARIs After Lockdown

During the second half of 2020, most viruses returned to or exceeded historical levels. For many of them an off-season increase has been observed. Most available studies on the re-emergence of viral infections after lockdown concerned RSV infection [71,72].

#### 3.3.1. Respiratory Syncytial Virus

The reappearance of RSV after the strict lockdown has been described worldwide [71,72,73,74,75,76,77,78], targeting a wide immunologically naïve population largely consisting of infants born from mothers who did not reinforce their immunity to RSV [35,79,80]. However, the emergence of RSV epidemics appeared in different seasons and on a different scale compared to previous country-by-country trends [81,82,83,84,85]. These differences may be explained by factors such as climate, culture, quarantine methods, personal protective equipment, hygiene measures, and habits [71]. Nevertheless, the possibility that adults act as a reservoir of infection, significantly impacting RSV epidemiology, has been hypothesized [86]. Furthermore, a persistent negative impact of the pandemic on health-care systems in middle-income regions has been recently hypothesized to explain why the rebound in hospitalization rates of RSV-associated ARIs was mainly limited to high-income countries [87].

An increase in RSV cases at the end of winter 2020 and in the early spring was observed throughout New York state and suggested interseasonal RSV resurgence associated with the reduction of COVID-19 preventive efforts [88], as observed in other countries [89,90,91].

In Taiwan, an unexpected outbreak of bronchiolitis occurred between September and December 2020, even though in this country the pre-pandemic peak of bronchiolitis was usually observed in spring and summer [92].

In Spain, schools reopened in September 2020, when bronchiolitis and RSV cases were expected to re-appear [30]. However, despite re-opening schools, cases of bronchiolitis remained limited. Even more surprisingly, RSV diagnoses were nearly zero at least until the end of 2020 [30]. One possible explanation is that SARS-CoV-2 may have displaced other respiratory viruses, probably replacing their ecological niche, with a disproportionate effect on RSV [30]. In Italy, cases of bronchiolitis increased significantly in October 2021, peaked in November and December, and declined rapidly during the second half of December [93,94,95,96]. The leading role of RSV within the resurgence of viral pathogens after COVID-19-related social distancing has been further confirmed, more recently in some studies analyzing pediatric ARIs during the fall–winter of 2022/2023 [97,98,99,100]. These findings, obtained in very different geographical settings, confirm the high relevance that RSV re-gained after significant suppression during the pandemic.

Regarding the age of the affected children, data from a French birth cohort including infants (<1 year of age) admitted to the hospital with respiratory symptoms due to RSV showed an increase in the median age of affected children compared to previous seasons (4.8 months in 2020–2021 compared to 2.2 and 3.1 months in the 2016–2017 and 2019–2020 seasons, respectively) [101]. The older age of RSV-infected children in 2020–2021 was confirmed in other countries [93,102,103,104,105,106]. Furthermore, a shorter duration of RSV epidemics was reported in 2020–2021 compared to pre-COVID-19 seasons both in the Southern and Northern hemispheres [93,107,108].

Data on the clinical severity of RSV in 2020–2021 are conflicting. It is unclear whether children with a first RSV infection at an older age may be less [109,110] or more prone [111,112,113] to a severe clinical presentation. Infants born in the pandemic years were born to mothers with no RSV exposure during pregnancy, and, since neutralizing antibodies to RSV are short-lived, no significant mother-to-child transmission of RSV-specific antibodies can be expected [113]. This would have made it reasonable to expect a more severe clinical presentation of bronchiolitis. In a study from the US, 81% of children hospitalized with bronchiolitis were admitted to the PICU [92]. These data were confirmed by studies from Italy, Mexico, and South Korea reporting moderate to severe symptoms in approximately 50% of admitted patients [114,115,116]. More importantly, another study showed that almost twice as many patients with bronchiolitis were admitted to the PICU during the post-lockdown phase compared to the usual winter spike in cases (in the pre-lockdown period) [117]. Furthermore, the post-lockdown group was older at admission [117,118,119], but no differences in gender or ethnicity were reported [118]. Conversely, national data from New Zealand for children aged 0–4 years showed an increase in PICU discharges for bronchiolitis in 2021, which was 2.8 times higher than the average recorded in 2015–2019 [120]. However, this increase reflects an incidence rate of hospitalized bronchiolitis of 284 per 100,000 children in 2021, which was three times higher than the average of peaks in 2015–2019 [120]. Therefore, these similar increases in the rates of hospitalization and PICU discharges suggests that, despite a higher incidence of the disease, clinical presentation was not more severe than in previous years.

With regard to RSV genomic diversity in the post-COVID-19 period, an Australian study analyzed genome sequencing on RSV-positive specimens collected before (July 2017–March 2020), during, and after (April 2020–March 2021) the implementation of COVID-19 restrictions [121]. The authors found a variable prevalence of the pre-pandemic co-circulation of RSV-A and B subtypes, with RSV-A accounting for 45–79% of cases. Between late 2020 and early 2021 there was a predominance of the RSV-A subtype (>95%), suggesting that RSV-A was the sole sub-type responsible for 2020 RSV outbreaks [122]. In addition, the analysis of the G gene phylogeny demonstrated that RSV circulating during the Australian 2020–2021 epidemic did not cluster with any other RSV virus observed before pandemic. This finding, in line with similar studies carried out in other countries [122,123,124], suggests that pre-pandemic RSV-A and B were largely eliminated during the COVID-19 restrictions allowing the circulation of new lineages [121,125,126].

Similarly, genomic data from pre- and post-pandemic seasons have also shown a shift in the predominant subtype from RSV-A to RSV-B in European and non-European countries [127,128,129]. Indeed, even though with variations in the considered periods among the available studies, during the 2021 season there was a dominance of RSV-A, whereas in 2022/23 RSV-B was more prevalent [127,128,129,130,131].

#### 3.3.2. Influenza

A study from China including a large cohort of pediatric and adult inpatients and outpatients with or without respiratory symptoms showed no significant peak in IFV-A and IFV-B between December 2020 and February 2021 [52]. Nevertheless, during the flu season of 2021–2022, IFV-B emerged as a leading cause of respiratory infections [132]; a broad surveillance program involving adults and children from two cities in Eastern China detected both IFV-A and IFV-B as the most frequent viral pathogens in the 2023 fall–winter season [133]. Similarly, data from several European and non-European countries showed that since April 2021, IFV-A and B increased disproportionately and reached a peak varying between October 2021 and early 2022 [134,135,136,137], even though the higher number of hospitalizations was not apparently associated with higher severity or mortality [138,139].

With regard to IFV subtypes, the pseudo-extinction of B/Yamagata lineage was reported globally during the pandemic [140,141], with no evidence of resumed circulation after the relaxation of COVID-19 control measures [142]. The implications of this shift, likely deriving from differences in susceptible populations, are potentially immense [142]. Nevertheless, a significant impact on vaccination strategies has already been determined with the recent exclusion of a B/Yamagata antigen from the influenza vaccine [143].

#### 3.3.3. Other Viruses

In several studies, HRV outbreaks were reported both in adults and children both immediately after the re-opening of schools [18,144,145,146,147], and, more recently, during the fall of 2023 [148,149]. Reported outbreaks not only suggest that the virus has spread from schools to the broader community, but indicate that, despite occasional detection in children with no or mild respiratory symptoms, HRV may be associated with severe clinical presentation, with a need for oxygen supplementation in a significant proportion of cases [148,150,151]. The impact of the COVID-19 pandemic on HRV epidemiology has been further documented in South Korea, where schools were closed in March and re-opened in May 2020, while the authorities continued to minimize crowding and maintain face masks and hand hygiene policies [152,153]. In such settings, HRV surged after schools reopened and decreased only after the intensification of social distancing rules [152]. These data suggest that early social distancing measures reduced exposure to HRV during the typical transmission season (January–March), resulting in attenuated acquired immunity and a delayed surge in the fall when social distancing was relaxed. It is notable that, despite the large HRV resurgence, transmission of SARS-CoV-2 remained low in South Korean school-aged children, indicating different methods of transmission [154]. In a study from Finland, the increase in the incidence of HRV in children began in early summer 2020, when school-aged children and some day-care-aged children were having summer holidays [155]. The increase in the summer was likely due to the lifting of social restrictions aimed towards the adult population, as bars were opened and restrictions for gatherings were eased [155].

Although far less studied in comparison to other viruses, data on human metapneumovirus (hMPV) transmission throughout the pandemic years were reported by an Australian group that confirmed the susceptibility of such pathogens to the non-pharmacological mitigation measures adopted to combat COVID-19 [156,157]. The authors found that, after a substantial absence in 2020, hMPV incidence in 2021 almost tripled compared to 2017–2019, with children aged 1–4 years being most frequently affected. As with other viruses, the post-lockdown increased incidence was likely linked to a wider cohort of hMPV-naïve children and waning population immunity [156].

## 4. Discussion

A significant impact of the COVID-19 pandemic on the circulation of common respiratory viruses has been observed worldwide. The non-pharmacological measures implemented during the pandemic deeply modified the usual epidemiology of common childhood respiratory infections, ultimately leading to a significant decrease in the numbers of pediatric hospital admissions because of ARIs compared to non-pandemic years [158,159]. This reduction could be considered to be an unexpected positive consequence of the non-pharmacological measures taken during the COVID-19 pandemic. Another possible explanation is parents’ reluctance to take their children to healthcare facilities during the pandemic due to fear of SARS-CoV-2 exposure, which could have partially contributed to the decline in the number of patients diagnosed with ARIs [160,161]. Importantly, and most relevant for RSV, during the 2020–2021 season in Europe, when RSV activity was overall very low, the only countries with major RSV outbreaks were those whose policies were to keep primary schools and childcare centers opened throughout lockdowns [162].

When COVID-19 mitigation practices became less stringent—and especially with the re-opening of schools—outbreaks of respiratory infections were registered almost worldwide [71,72,73,74,75,76,77,78,134,135,136,137,144,145,146,147]. With the reoccurrence of viral infections, most episodes were registered outside of the typical pre-pandemic season and in an unexpected age range [81,82,83,84,85,102,103,104,105,106]. The mechanism behind this off-season resurgence is still unclear. Furthermore, the resurgence of respiratory viruses followed different dynamics depending on the type of pathogen. For example, IFV and RSV did not produce a seasonal epidemic in the 2020–21 winter season, while other pathogens such as HRV circulated at various levels and intensity [163]. Factors that determine the spread of pathogens include virological features (virulence, fitness, and transmissibility) and immune evasion, as well as seasonal variations, host characteristics (age, co-morbidities, asymptomatic viral carriage, personal hygiene, and proximity to other hosts), and environmental conditions (temperature, humidity, and the contamination of surfaces) [164,165,166,167,168]. In addition to mitigation due to COVID-19 control measures, the reduction in influenza vaccination campaigns may be another important component of the resurgence in respiratory infections.

The most important differences in outbreaks after lockdown were observed for RSV. Even in the same hemisphere, country-by-country differences in its resurgence in the 2020–2021 season were reported [30,93]. In most countries, schools reopened beginning in September 2020. However, opportunities for viral transmission outside of school from other children or within the family remained. Differences in viruses’ circulation may have been due, at least in part, to restrictive measures to contain SARS-CoV-2 outside of school, which differed by country. For example, in the Campania region (southern Italy), measures to control the spread of SARS-CoV-2 were longer and stricter compared to other Italian regions and to other European countries. The lockdown required the closure of all grade schools and the restriction of children’s group activities from March to October 2020. In the following months (between the end of 2020 and the beginning of 2021), schools remained closed again for a long period in response to SARS-CoV-2 regional peaks. Therefore, during the first two years of the pandemic, there were very few cases of children hospitalized because of ARIs due to RSV or other viruses [36]. With the re-opening of social activities, there was an unexpected peak of respiratory infections, emergency department visits and hospital admissions at one pediatric hospital [93]. Another explanation for the discrepancies in peaks between different countries is the change in testing practices, with more systematic tests performed due to the COVID-19 pandemic, including more tests, even for RSV, in older children [113].

Concerns have been raised about potential future epidemics from respiratory viruses, particularly RSV, within pediatric communities that do not encounter these pathogens over a period of two years and are at risk of paying the so-called “immunity debt” [12]. Available data on the clinical severity of respiratory infections in the post-lockdown period are conflicting [109,110,111,112,113]. On the one hand, increased age at the time of initial infection may be expected to entail reduced hospitalizations, given that the RSV burden is most pronounced in neonates and young infants. On the other hand, this was not reflected in a reduction in the number of bronchiolitis cases admitted, at least in some countries [121]. It may be assumed that, by increasing the pool of susceptible children, including those with underlying risk factors, outbreaks might be more severe with regard to hospitalizations and PICU admissions.

The increased age of RSV infected children following the start of the COVID-19 pandemic is noteworthy [101,102,103,104,105,106]. Several explanations have been hypothesized. Due to the absence of RSV activity following the start of the COVID-19 pandemic, a larger number of infants and young children (with a slight upward age shift) were at increased risk for severe RSV infection because they did not have the opportunity to develop immunity against this pathogen.

Nevertheless, discrepancies in the results from different studies prevent a firm conclusion. Indeed, from an epidemiological point of view, not all the studies had the availability of a program of nationwide surveillance for respiratory infections [35,47,118], and sentinelled data were often used [18,22,23]. Secondly, different methodologies were applied, including many retrospective studies [36,48,75,76,149] and a limited minority of prospective observations [57,65,101]. Moreover, the COVID-19 era changed institutional testing strategies compared to previous seasons, with more extensive testing for viruses such as RSV in older children as well [113]. Finally, RSV epidemiology is currently undergoing a revolution with the potential of completely changing the impact of such pathogen on clinical practice: the widespread administration of nirsevimab, the novel monoclonal antibody against RSV, to neonates and infants [169]. Such an intervention, despite varying by geographic area with regard to the start season and the number of immunized children, is likely to dramatically decrease RSV circulation, with an extraordinary impact in terms of saved lives and reduced hospitalizations. Whether this change will entail the increased diffusion of other viral pathogens that will replace RSV in seasonal epidemics is still to be determined and will represent a topic of major interest in the next few years.

The present review has several limitations. First, despite being extended to a significant number of articles, the literature search was limited to three databases and to studies with full text availability. This aspect, together with the huge number of articles screened, may have resulted in the exclusion of some potentially relevant studies. Furthermore, a comprehensive analysis of the genetic characteristics of the respiratory viruses, specifically addressing their changes during and after the COVID-19 pandemic, was not performed. The reported details derive from the few studies included in the selection process, but are far from exhaustive, as a specific literature search or analysis of genomic databases was not carried out.

## 5. Conclusions

The strict public health measures adopted to combat the COVID-19 pandemic were successful in limiting community transmission of SARS-CoV-2. In addition, these measures resulted in a significant reduction in infections caused by other respiratory viruses, with a consequent reduction in childhood morbidity, including hospital and PICU admissions. On the other hand, the lockdown measures, which effectively resulted in COVID-19 containment, were very restrictive of personal liberties and, in the absence of a pandemic threatening lives and hospital systems, would not be considered reasonable by the general community. After these dramatic public health changes, the characteristics of viral epidemics and their mutations should be extensively and continuously monitored and analyzed. It remains unclear how long it will take for the seasonality of normal winter respiratory viruses (mainly RSV and IFV) to resume globally. Considering the yearly variations in viral infections’ peaks and rates of infection, it is uncertain whether off-season or more severe respiratory infections will occur in the near future.

Future public health strategies should include the implementation of monitoring systems for respiratory viruses, including a careful assessment of the trends and patterns of circulation and co-infection among children. Such a tool could be crucial for providing timely interventions when new outbreaks occur and may guide preventive strategies aimed at limiting future epidemics, such as non-pharmacological measures or anti-viral prophylaxis in high-risk groups. Further research is also needed to better understand which public health interventions are most effective in reducing the diffusion of the different viral pathogens and when these interventions should be applied to obtain the best balance between prevention of viral spread and social acceptability. Finally, further advances in the link between the viral genome, viral phenotype, and viral interaction with host immunity will hopefully pave the way towards the development of new vaccines, particularly aimed at limiting infections in subjects with underlying conditions or with immunocompromised status.

## Data Availability

The data presented in this study are available on request from the corresponding author.

## Supplementary Materials

The following supporting information can be downloaded at: https://www.mdpi.com/article/10.3390/jcm14041387/s1, Table S1. Main characteristics of the 148 articles included in the Review; Table S2. Articles initially included during the literature screening but then excluded from the final selection.
