# Supplementary material for: Changes in Respiratory Viruses’ Activity in Children During the COVID-19 Pandemic: A Systematic Review
Source: J Clin Med. 2025 Feb 19;14(4):1387. doi: 10.3390/jcm14041387 (PMC11856189; doi:10.3390/jcm14041387)
Supplement: Supplementary file 1 [file jcm-14-01387-s001.zip › jcm-3367824-supplementary.pdf]

Supplementary Table 1. Main characteristics of the 148 articles included in the Review.

No.	Ref. No.	First Author	Title	Journal	Year	Study design	Tested viruses	Country	Study quality
1	8	Furuse, Y.	Epidemiological and clinical characteristics of children with acute respiratory viral infections in the Philippines: a prospective cohort study.	*Clin. Microbiol. Infect.*	2021	Prospective cohort study	ADV, EV, HMPV, IFV, PIV, HRV, RSV	Philippines	high
2	9	Jefferson, T.	Physical interventions to interrupt or reduce the spread of respiratory viruses.	*Cochrane Database Syst. Rev.*	2020	Review	-	United Kingdom	-
3	10	Britton, P.N.	COVID-19 public health measures and respiratory syncytial virus.	*Lancet Child Adolesc. Health*	2020	Retrospective, observational	RSV	Australia	high
4	11	Angoulvant, F.	Coronavirus Disease 2019 Pandemic: Impact Caused by School Closure and National Lockdown on Pediatric Visits and Admissions for Viral and Nonviral Infections-a Time Series Analysis.	*Clin. Infect. Dis.*	2021	Retrospective, observational	-	France	moderate

5	12	Cohen, R.	Pediatric Infectious Disease Group (GPIP) position paper on the immune debt of the COVID-19 pandemic in childhood, how can we fill the immunity gap?	*Infect. Dis. Now*	2021	Review	-	France	-
6	15	Guadalupe-Fernández, V.	Epidemiological Surveillance Network of Catalonia. Investigating epidemiological distribution (temporality and intensity) of respiratory pathogens following COVID-19 de-escalation process in Catalonia, September 2016-June 2021: Analysis of regional surveillance data.	*PLoS One*	2024	Retrospective, observational data analysis	ADV, IFV, PIV, RSV	Spain	moderate
7	16	Li, Z.J.	Broad Impacts of Coronavirus Disease 2019 (COVID-19) Pandemic on Acute Respiratory Infections in China: An Observational Study.	*Clin. Infect. Dis.*	2022	Retrospective, observational	ADV, IFV, RSV, PIV, HMPV, HCoV, HBoV, HRV	China	moderate
8	17	Armero, G.	Non-Pharmacological Interventions During SARS-CoV-2 Pandemic:	*Arch. Bronconeumol.*	2024	Retrospective, observational	ADV, IFV, RSV, PIV, HMPV, HCoV, HRV	Spain	moderate

			Effects on Pediatric Viral Respiratory Infections.						
9	18	Li, Y.	Prevalence of respiratory viruses among hospitalized children with lower respiratory tract infections during the COVID-19 pandemic in Wuhan, China.	*Int. J. Infect. Dis.*	2024	Retrospective, observational	ADV, IFV, RSV, PIV	China	moderate
10	19	Zhang, G.	Epidemiological changes in respiratory pathogen transmission among children with acute respiratory infections during the COVID-19 pandemic in Kunming, China.	*B.M.C. Infect. Dis.*	2024	Prospective, observational	ADV, IFV, RSV, PIV, HMPV, HCoV, HBoV, HRV	China	moderate
11	20	Shen, D.P.	Impact of COVID-19 on viral respiratory infection epidemiology in young children: A single-center analysis.	*Front. Public Health*	2022	Retrospective, observational	ADV, IFV, RSV, PIV, HMPV, HCoV, HBoV, HRV	Belgium	moderate
12	21	Ma, J.E.	Analysis of Common Respiratory Infected Pathogens in 3100 Children after the Coronavirus Disease 2019 Pandemic.	*Curr. Med. Sci.*	2022	Retrospective, observational	ADV, IFV, RSV, PIV	China	moderate

13	22	Wu, R.	Analysis of respiratory virus detection in hospitalized children with acute respiratory infection during the COVID-19 pandemic.	*Virol. J.*	2023	Retrospective, observational	ADV, IFV, RSV, PIV	China	moderate
14	23	De Maio, F.	Respiratory viruses in the pre and post-pandemic periods in an Italian tertiary hospital.	*Immun. Inflamm. Dis.*	2023	Retrospective, observational	ADV, IFV, RSV, PIV, HMPV, HCoV, HBoV, HRV	Italy	moderate
15	24	Baldassarre, M.E.	Hospitalization for bronchiolitis in children aged ≤ 1year, Southern Italy, year 2021: need for new preventive strategies?	*Ital. J. Pediatr.*	2023	Retrospective, observational	RSV	Italy	moderate
16	25	Chuang, Y.C.	The Impact of the COVID-19 Pandemic on Respiratory Syncytial Virus Infection: A Narrative Review.	*Infect. Drug Resist.*	2023	Review	-	Taiwan	-
17	26	Haddadin, Z.	Acute Respiratory Illnesses in Children in the SARS-CoV-2 Pandemic: Prospective Multicenter Study.	*Pediatrics*	2021	Prospective, observational	ADV, IFV, RSV, PIV, HMPV, HCoV, HBoV, HRV	USA	moderate
18	27	Curatola, A.	Impact of COVID-19 outbreak in acute bronchiolitis: Lesson	*Pediatr. Pulmonol.*	2021	Retrospective, observational	ADV, IFV, RSV, PIV, HMPV, HRV	Italy	moderate

			from a tertiary Italian Emergency Department.						
19	28	Guedj, R.	Infant bronchiolitis dramatically reduced during the second French COVID-19 outbreak.	*Acta Paediatr.*	2021	Retrospective, observational	-	France	moderate
20	29	Friedrich, F.	Early Impact of Social Distancing in Response to Coronavirus Disease 2019 on Hospitalizations for Acute Bronchiolitis in Infants in Brazil.	*Clin. Infect. Dis.*	2021	Retrospective, observational	-	Brazil	moderate
21	30	Torres-Fernandez, D.	Acute bronchiolitis and respiratory syncytial virus seasonal transmission during the COVID-19 pandemic in Spain: A national perspective from the pediatric Spanish Society (AEP).	*J. Clin. Virol.*	2021	Retrospective, observational	RSV	Spain	moderate
22	31	Van Brusselen, D.	Bronchiolitis in COVID-19 times: a nearly absent disease?	*Eur. J. Pediatr.*	2021	Retrospective, observational	RSV	Belgium	moderate
23	32	Razanajatovo, N.H.	Epidemiological Patterns of Seasonal Respiratory Viruses during the COVID-19	*Viruses*	2022	Retrospective, observational	IFV, RSV	Madagascar	high

			Pandemic in Madagascar, March 2020-May 2022.						
24	33	Bhardwaj, S.	Resurgence of respiratory syncytial virus infection during COVID-19 pandemic in Pune, India.	*B.M.C. Infect. Dis.*	2024	Retrospective, observational	RSV	India	high
25	34	Yang, M.C.	Changing patterns of infectious diseases in children during the COVID-19 pandemic.	*Front. Cell Infect. Microbiol.*	2023	Review	-	Taiwan	-
26	35	Bardsley, M.	Epidemiology of respiratory syncytial virus in children younger than 5 years in England during the COVID-19 pandemic, measured by laboratory, clinical, and syndromic surveillance: a retrospective observational study.	*Lancet Infect. Dis.*	2023	Retrospective, observational	RSV	England	high
27	36	Berdah, L.	Retrospective observational study of the influence of the COVID-19 outbreak on infants' hospitalisation for acute bronchiolitis.	*B.M.J. Open*	2022	Retrospective, observational	IFV, RSV	France	high

28	37	Bermúdez Barrezueta, L.	Influence of the COVID-19 pandemic on the epidemiology of acute bronchiolitis.	*Enferm. Infecc. Microbiol. Clin. (Engl. Ed.)*	2023	Retrospective /prospective, observational	ADV, IFV, RSV, PIV, HMPV, HCoV, HRV	Spain	moderate
29	38	Jiang, X.	Clinical characteristics and etiology of children with bronchiolitis before and during the COVID-19 pandemic in Suzhou, China.	*Front. Pediatr.*	2022	Retrospective, observational	ADV, IFV, RSV, PIV,	China	moderate
30	39	Kıymet, E.	Distribution of spreading viruses during COVID-19 pandemic: Effect of mitigation strategies.	*Am. J. Infect. Control*	2021	Retrospective, observational	ADV, IFV, RSV, PIV, HMPV, HCoV, HBoV, HRV, PEV	Turkey	high
31	40	Lucion, M.F.	Impact of COVID-19 on the circulation of respiratory viruses in a children's hospital: an expected absence.	*Arch. Argent. Pediatr.*	2022	Retrospective, observational	ADV, IFV, RSV, PIV,	Argentina	moderate
32	42	Rambaud, J.	Bronchiolitis Admissions to Intensive Care During COVID.	*Pediatrics*	2021	Retrospective, observational	-	France	moderate
33	43	Hassan, M.Z.	Respiratory Syncytial Virus-Associated Deaths among Children under Five before and during the COVID-19	*Viruses*	2024	Retrospective, observational	ADV, IFV, RSV, PIV, HMPV, SARS-CoV-2,	Bangladesh	moderate

			Pandemic in Bangladesh.						
34	44	Giannattasio, A.	Silent RSV in infants with SARS-CoV-2 infection: A case series.	*Pediatr. Pulmonol.*	2021	Retrospective, observational	RSV, SARS-CoV-2	Italy	moderate
35	46	Abo, Y.N.	COVID-19 public health measures and respiratory viruses in children in Melbourne.	*J. Paediatr. Child Health*	2021	Retrospective, observational	IFV, RSV, PIV,	Australia	moderate
36	47	Badar, N.	Unraveling influenza sentinel surveillance in Pakistan 2008-2024: Epidemiological insights during the pre and post pandemic period of COVID-19.	*J. Infect. Public Health*	2024	Retrospective, observational	IFV	Pakistan	moderate
37	48	Bhardwaj, S.	A retrospective analysis of respiratory virus transmission before and during the COVID-19 pandemic in Pune the western region of India.	*Front. Public Health*	2022	Retrospective, observational	ADV, HMPV, IFV, PIV, HRV, RSV	India	moderate
38	49	Eisen, A.K.A.	Low circulation of Influenza A and coinfection with SARS-CoV-2 among other respiratory viruses during the COVID-19	*Brazil. J. Med. Virol.*	2021	Retrospective, observational	ADV, IFV, HRV, SARS-CoV-2	Brazil	high

			pandemic in a region of southern Brazil						
39	50	Monamele, G.C.	The Detection of Influenza Virus Before and During the COVID-19 Pandemic in Cameroon.	*Influenza Other Respir. Viruses*	2024	Retrospective, observational	IFV, SARS-CoV-2	Cameroon	moderate
40	51	Knudsen, P.K.	The incidence of infectious diseases and viruses other than SARS-CoV-2 amongst hospitalised children in Oslo, Norway during the Covid-19 pandemic 2020-2021.	*J. Clin. Virol. Plus*	2022	Retrospective, observational	ADV, HMPV, IFV, HRV, RSV	Norway	moderate
41	52	Du, X.	Exploring the epidemiological changes of common respiratory viruses since the COVID-19 pandemic: a hospital study in Hangzhou, China.	*Arch. Virol.*	2021	Retrospective, observational	ADV, IFV, RSV	China	moderate
42	53	Lee, H.	Impact of Public Health Interventions on Seasonal Influenza Activity During the COVID-19 Outbreak in Korea.	*Clin. Infect. Dis.*	2021	Retrospective, observational	IFV	South Korea	moderate

43	54	Fukuda, Y.	Surveillance in hospitalized children with infectious diseases in Japan: Pre- and post-coronavirus disease 2019.	*J. Infect. Chemother.*	2021	Prospective, observational	ADV, HMPV, IFV, RSV	Japan	moderate
44	55	Zendehrouh, M.	Respiratory Viral Infections Among Children Hospitalized in a Great Referral Hospital in Iran During the Coronavirus Pandemic.	*Arch. Pediatr. Infect. Dis.*	2023	Cross-sectional	ADV, IFV, RSV, PIV, HMPV, HCoV, HBoV, SARS-CoV-2	Iran	moderate
45	56	Kang, M.	Paradigm shift of respiratory viruses causing lower respiratory tract infection in children during COVID-19 pandemic in India.	*J. Infect. Dev. Ctries*	2023	Retrospective, observational	ADV, IFV, RSV, PIV, HMPV, HRV, HBoV	India	moderate
46	57	Loevinsohn, G.	Respiratory viruses in rural Zambia before and during the COVID-19 pandemic.	*Trop. Med. Int. Health*	2022	Prospective, observational	ADV, IFV, RSV, PIV, HMPV, HRV, HCoV	Zambia	moderate
47	58	Al Kindi, H.	Time trend of respiratory viruses before and during the COVID-19 pandemic in severe acute respiratory virus infection in the	*Influenza Other Respir. Viruses.*	2023	Prospective, observational	ADV, IFV, RSV, PIV, HMPV, HCoV, HBoV, HRV, PEV	Oman	moderate

			Sultanate of Oman between 2017 and 2022.						
48	59	Umran, N.	Prevalence of non-SARS CoV-2 respiratory virus infection in children during COVID-19 pandemic in Chennai, South India.	*Indian J. Pathol. Microbiol.*	2024	Prospective, observational	ADV, IFV, RSV, PIV, HMPV, HRV,	India	moderate
49	60	Nenna, R.	First COVID-19 lockdown resulted in most respiratory viruses disappearing among hospitalised children, with the exception of rhinoviruses.	*Acta Paediatr.*	2022	Retrospective, observational	ADV, IFV, RSV, PIV, HMPV, HCoV, HBoV, HRV	Italy	moderate
50	61	Pun, J.C.S.	Respiratory Viral Infection Patterns in Hospitalised Children Before and After COVID-19 in Hong Kong.	*Viruses*	2024	Retrospective, observational	ADV, IFV, RSV, PIV, HRV	Hong Kong	moderate
51	62	Takashita, E.	Increased risk of rhinovirus infection in children during the coronavirus disease-19 pandemic	*Influenza Other Respir. Viruses*	2021	Retrospective, observational	IFV, HRV	Japan	moderate

52	63	Agca, H.	Changing epidemiology of influenza and other respiratory viruses in the first year of COVID-19 pandemic.	*J. Infect. Public Health*	2021	Retrospective, observational	ADV, IFV, RSV, HMPV, HCoV, HRV	Turkey	moderate
53	64	Kume, Y.	Changes in virus detection in hospitalized children before and after the severe acute respiratory syndrome coronavirus 2 pandemic.	*Influenza Other Respir. Viruses.*	2022	Retrospective, observational	ADV, IFV, RSV, PIV, HMPV, HCoV, HBoV, HRV,	Japan	moderate
54	65	Engels, G.	Incidence of SARS-CoV-2, Influenza and RSV but High Incidence of Rhino-, Adeno- and Endemic Coronaviruses in Children With Acute Respiratory Infection in Primary Care Pediatric Practices During the Second and Third Wave of the SARS-CoV-2 Pandemic.	*Pediatr. Infect. Dis. J.*	2022	Prospective, observational	ADV, IFV, RSV, PIV, HMPV, HCoV, HBoV, HRV, PEV	Germany	moderate
55	66	Vittucci, A.C.	The Disappearance of Respiratory Viruses in Children during the COVID-19 Pandemic.	*Int. J. Environ. Res. Public Health*	2021	Retrospective, observational	ADV, IFV, RSV, PIV, HMPV, HCoV, HBoV, HRV	Italy	moderate

56	67	Boyanton, B.L. Jr	SARS-CoV-2 pandemic non-pharmacologic interventions temporally associated with reduced pediatric infections due to *Mycoplasma pneumoniae* and co-infecting respiratory viruses in Arkansas.	*Microbiol. Spectr.*	2024	Retrospective, observational	ADV, IFV, RSV, PIV, HMPV, HCoV, HRV,	USA	moderate
57	68	Teo, K.W.	Rhinovirus persistence during the COVID-19 pandemic-Impact on pediatric acute wheezing presentations.	*J. Med. Virol.*	2022	Retrospective	ADV, RSV, PIV, HMPV, HRV	United Kingdom	moderate
58	69	Dallmeyer, L.K.	Epidemiology of respiratory viruses among children during the SARS-CoV-2 pandemic: A systematic review and meta-analysis.	*Int. J. Infect. Dis.*	2024	Meta-analysis	-	Germany	-
59	70	Arunasalam, S.	Demographic and clinical characteristics of human bocavirus-1 infection in patients with acute respiratory tract infections during the COVID-19	*B.M.C. Infect. Dis.*	2023	Prospective, observational	ADV, IFV, RSV, PIV, HMPV, HCoV, HBoV, HRV, SARS-CoV-2	Sri Lanka	moderate

			pandemic in the Central Province of Sri Lanka.						
60	71	Leija-Martínez, J.J.	Impact of Nonpharmaceutical Interventions during the COVID-19 Pandemic on the Prevalence of Respiratory Syncytial Virus in Hospitalized Children with Lower Respiratory Tract Infections: A Systematic Review and Meta-Analysis.	*Viruses*	2024	Meta-analysis	-	Mexico	-
61	72	Davids, M.	Changes in Prevalence and Seasonality of Pathogens Identified in Acute Respiratory Tract Infections in Hospitalised Individuals in Rural and Urban Settings in South Africa; 2018-2022.	*Viruses*	2024	Retrospective, observational	ADV, IFV, RSV, PIV, HMPV, HCoV, HBoV, HRV, PEV	South Africa	moderate
62	73	Wang, H.	Lockdown measures during the COVID-19 pandemic strongly impacted the circulation of respiratory pathogens in Southern China.	*Sci. Rep.*	2022	Retrospective, observational	ADV, IFV, RSV, PIV, HMPV, HCoV, HBoV, HRV	Southern China	moderate

63	74	Fafi, I.	Evolution of respiratory syncytial virus burden in young children following the COVID-19 pandemic: influence of concomitant changes in testing practices.	*Lancet Infect. Dis.*	2024	Retrospective, observational	RSV	France	moderate
64	75	Gashgarey, D.	Evolving Epidemiology of Pediatric Respiratory Syncytial Virus (RSV) Cases Around COVID-19 Pandemic: Impact and Clinical Insights, Retrospective Cohort Study.	*J. Epidemiol. Glob. Health*	2024	Retrospective, observational	RSV	Saudi Arabia	moderate
65	76	Nixon, J.C.	Altered epidemiological patterns of Respiratory Syncytial Virus and influenza detections in a tropical Australian setting 2020 to 2023.	*Aust. N. Z. J. Public Health*	2024	Retrospective, observational	RSV, IFV, SARS-CoV-2	Australia	moderate
66	77	Noble, M.	Respiratory syncytial virus-associated hospitalisation in children aged ≤5 years: a scoping review of literature from 2009 to 2021.	*E.R.J. Open Res.*	2022	Review	-	United Kingdom	-
67	78	Bedir Demirdag, T.	Distribution and clinical features of viral	*J. Pediatr. Inf.*	2022	Retrospective, observational	ADV, IFV, RSV, PIV, HMPV,	Turkey	moderate

			respiratory infections in children after face-to-face education in 2021-2022 winter period.				HCoV, HBoV, HRV, PEV		
68	79	Di Mattia, G.	During the COVID-19 pandemic where has respiratory syncytial virus gone?	*Pediatr. Pulmonol.*	2021	Review	-	Italy	-
69	80	Jiang, W.	Exploring immunity debt: Dynamic alterations in RSV antibody levels in children under 5 years during the COVID-19 pandemic.	*J. Infect.*	2024	Retrospective /prospective, observational	-	China	moderate
70	81	Mazela, J.	Epidemiology of Respiratory Syncytial Virus Hospitalizations in Poland: An Analysis from 2015 to 2023 Covering the Entire Polish Population of Children Aged under Five Years.	*Viruses*	2024	Retrospective, observational	RSV	Poland	moderate
71	82	Kahanowitch, R.	How did respiratory syncytial virus and other pediatric respiratory viruses change during the COVID-19 pandemic?	*Pediatr. Pulmonol.*	2022	Retrospective, observational	ADV, IFV, RSV, PIV, HMPV, HRV,	USA	moderate

72	83	Faraguna, M.C.	The bronchiolitis epidemic in 2021-2022 during the SARS-CoV-2 pandemic: experience of a third level centre in Northern Italy.	*Ital. J. Pediatr.*	2023	Retrospective, observational	RSV	Italy	moderate
73	84	Wu, Y.	Epidemiological study of post-pandemic pediatric common respiratory pathogens using multiplex detection.	*Virol. J.*	2024	Retrospective, observational	ADV, IFV, RSV, PIV,	China	high
74	85	Nenna, R.	An Italian Multicenter Study on the Epidemiology of Respiratory Syncytial Virus During SARS-CoV-2 Pandemic in Hospitalized Children.	*Front. Pediatr.*	2022	Prospective, observational	ADV, IFV, RSV, PIV, HMPV, HRV	Italy	high
75	86	Binns, E.	Influenza and respiratory syncytial virus during the COVID-19 pandemic: Time for a new paradigm?	*Pediatr. Pulmonol.*	2022	Commentary	-	-	-
76	87	Cong, B.	Changes in the global hospitalisation burden of respiratory syncytial virus in young children during the COVID-19	*Lancet Infect. Dis.*	2024	Systematic review	-	-	-

			pandemic: a systematic analysis.						
77	88	Halabi, K.C.	The Epidemiology of Respiratory Syncytial Virus in New York City during the Coronavirus Disease-2019 Pandemic Compared with Previous Years.	*J. Pediatr.*	2022	Retrospective, observational	RSV	USA	moderate
78	89	Foley, D.A.	The Interseasonal Resurgence of Respiratory Syncytial Virus in Australian Children Following the Reduction of Coronavirus Disease 2019-Related Public Health Measures.	*Clin. Infect. Dis.*	2021	Retrospective, observational	RSV	Australia	moderate
79	90	Weinberger Opek, M.	Delayed respiratory syncytial virus epidemic in children after relaxation of COVID-19 physical distancing measures, Ashdod, Israel, 2021.	*Euro Surveill.*	2021	Retrospective, observational	RSV	Israel	moderate
80	91	Agha, R.	Delayed Seasonal RSV Surge Observed During the COVID-19 Pandemic.	*Pediatrics*	2021	Retrospective, observational	RSV	USA	moderate

81	92	Hsu, H.T.	The epidemiological features of pediatric viral respiratory infection during the COVID-19 pandemic in Taiwan.	*J. Microbiol. Immunol. Infect.*	2021	Retrospective, observational	ADV, IFV, RSV, PIV,	Taiwan	moderate
82	93	Maglione, M.	Changing Epidemiology of Acute Viral Respiratory Infections in Hospitalized Children: The Post-Lockdown Effect.	*Children*	2022	Retrospective, observational	ADV, IFV, RSV, PIV, HMPV, HCoV, HRV, MERS, SARS-CoV-2	Italy	moderate
83	94	Camporesi, A.	Epidemiology, Microbiology and Severity of Bronchiolitis in the First Post-Lockdown Cold Season in Three Different Geographical Areas in Italy: A Prospective, Observational Study.	*Children*	2022	Prospective, observational	ADV, IFV, RSV, PIV, HMPV, HRV, HBoV	Italy	high
84	95	Manti, S.	Impact of COVID-19 Pandemic and Lockdown on the Epidemiology of RSV-Mediated Bronchiolitis: Experience from Our Centre.	*Children*	2022	Retrospective, observational	RSV	Italy	moderate
85	96	Treggiari, D.	Characteristics of Respiratory Syncytial	*Viruses*	2024	Retrospective, observational	IFV, RSV, SARS-CoV-2	Italy	moderate

			Virus Infections in Children in the Post-COVID Seasons: A Northern Italy Hospital Experience.						
86	97	Stacevičienė, I.	Epidemiological changes of acute respiratory infections in children: A single-center experience after COVID-19 lockdown.	*PLoS One*	2024	Retrospective, observational	IFV, RSV, SARS-CoV-2	Lithuania	moderate
87	98	Falsaperla, R.	Acute Respiratory Tract Infections (ARTIs) in Children after COVID-19-Related Social Distancing: An Epidemiological Study in a Single Center of Southern Italy.	*Diagnostics*	2024	Retrospective, observational	ADV, IFV, RSV, PIV, HMPV, HCoV, HRV, MERS, SARS-CoV-2	Italy	moderate
88	99	Parola, F.	Impact of SARS-CoV-2 Pandemic and Lockdown on the HRSV Circulation: Experience of Three Spoke Hospitals in Northern Italy.	*Viruses*	2024	Retrospective, observational	RSV	Italy	high
89	100	Lima, A.K.S.	Seasonal respiratory virus trends in pediatric patients during the	*Braz. J. Microbiol.*	2023	Retrospective, observational	ADV, IFV, RSV, HRV, SARS-CoV-2	Brazil	moderate

			COVID-19 pandemic in Brazil.						
90	101	Casalegno, J.S.	Characteristics of the delayed respiratory syncytial virus epidemic, 2020/2021, Rhône Loire, France.	*Euro Surveill.*	2021	Prospective, observational	RSV	France	high
91	102	Yeoh, D.K.	Impact of Coronavirus Disease 2019 public health measures on detections of influenza and respiratory syncytial virus in children during the 2020 Australian winter.	*Clin. Infect. Dis.*	2021	Retrospective, observational	IFV, RSV	Australia	moderate
92	103	McNab, S.	Changing Epidemiology of Respiratory Syncytial Virus in Australia-Delayed Re-Emergence in Victoria Compared to Western Australia/New South Wales (WA/NSW) After Prolonged Lock-Down for Coronavirus Disease 2019 (COVID-19).	*Clin. Infect. Dis.*	2021	Retrospective, observational	RSV	Australia	moderate
93	104	Fischli K	Postpandemic fluctuations of regional respiratory syncytial virus hospitalization	*Eur. J. Pediatr.*	2024	Retrospective, observational	RSV	Switzerland	moderate

			epidemiology: potential impact on an immunization program in Switzerland.						
94	105	Ren, L.	Epidemiological and clinical characteristics of respiratory syncytial virus and influenza infections in hospitalized children before and during the COVID-19 pandemic in Central China.	*Influenza Other Respir. Viruses*	2023	Prospective, case-series	IFV, RSV	China	moderate
95	106	Zheng, Z.	Estimation of the Timing and Intensity of Reemergence of Respiratory Syncytial Virus Following the COVID-19 Pandemic in the US.	*J.A.M.A. Netw. Open*	2021	Simulation modeling study	RSV	USA	moderate
96	109	Trigueros Montes, J.B.	The Impact of COVID-19 Pandemic on Respiratory Syncytial Virus Infection in Children.	*Pulm. Med.*	2024	Retrospective, observational	RSV	USA	moderate
97	110	Jiang, W.	Are we ready to face the next wave of RSV surge after the COVID-19 Omicron pandemic in China?	*Front. Cell Infect. Microbiol.*	2023	Prospective observational	ADV, IFV, RSV, HRV, HMPV	China	moderate

98	111	Mori, T.	Risk of admission requirement among children with respiratory infection in the post-COVID-19 pandemic era.	*J. Infect. Public Health*	2024	Retrospective, observational	ADV, IFV, RSV, PIV, HRV, HMPV, HCoV, SARS-CoV-2	Japan	moderate
99	112	Kim, Y.K.	Shift in Clinical Epidemiology of Human Parainfluenza Virus Type 3 and Respiratory Syncytial Virus B Infections in Korean Children Before and During the COVID-19 Pandemic: A Multicenter Retrospective Study.	*J. Korean Med. Sci.*	2022	Retrospective, observational	RSV, PIV	South Korea	moderate
100	113	Rao, S.	Shifting Epidemiology and Severity of Respiratory Syncytial Virus in Children During the COVID-19 Pandemic.	*J.A.M.A. Pediatr.*	2023	Retrospective, observational	RSV, IFV, SARS-CoV-2	USA	high
101	114	Brisca, G.	How has the SARS-CoV-2 pandemic changed the epidemiology and management of acute bronchiolitis?	*Pediatr. Pulmonol.*	2023	Retrospective, observational	RSV	Italy	moderate

102	115	Gastelum-Bernal, M.A.	Severity and complications in infants with respiratory syncytial virus infection after the SARS-CoV-2 pandemic.	*Bol. Med. Hosp. Infant Mex.*	2024	Prospective, observational	RSV	Mexico	moderate
103	116	Kim, Y.E.	Clinical Characteristics and Severity of Respiratory Syncytial Virus Infection in Korean Children during the Post-COVID-19 Pandemic Period.	*Infect. Chemother.*	2024	Retrospective, observational	RSV	South Korea	moderate
104	117	Cardenas, J.	Changes in Critical Bronchiolitis After COVID-19 Lockdown.	*Cureus*	2022	Retrospective, observational	ADV, IFV, RSV, PIV, HRV, HMPV, HCoV, SARS-CoV-2	USA	moderate
105	118	Pruccoli, G.	The Importance of RSV Epidemiological Surveillance: A Multicenter Observational Study of RSV Infection during the COVID-19 Pandemic.	*Viruses*	2023	Retrospective, observational	RSV, SARS-CoV-2	Italy	high
106	119	Zven, S.	Predicting the RSV Surge: Pediatric RSV Patterns of the COVID Pandemic.	*Pediatr. Infect. Dis. J.*	2023	Retrospective, observational	RSV	USA	moderate

107	121	Eden, J.S.	Off-season RSV epidemics in Australia after easing of COVID-19 restrictions.	*Nat. Commun.*	2022	Retrospective, observational	RSV	Australia	high
108	122	Virant, M.J.	Changes in HRSV Epidemiology but Not Circulating Variants in Hospitalized Children due to the Emergence of SARS-CoV-2.	*Viruses*	2023	Retrospective, observational	ADV, IFV, RSV, PIV, HRV, HMPV, HCoV, PEV	Slovenia	moderate
109	123	Dolores, A.	RSV reemergence in Argentina since the SARS-CoV-2 pandemic.	*J. Clin. Virol.*	2022	Retrospective, observational	RSV	Argentina	moderate
110	124	Hönemann, M.	In-Depth Analysis of the Re-Emergence of Respiratory Syncytial Virus at a Tertiary Care Hospital in Germany in the Summer of 2021 after the Alleviation of Non-Pharmaceutical Interventions Due to the SARS-CoV-2 Pandemic.	*Viruses*	2023	Retrospective, observational	ADV, IFV, RSV, PIV, HRV, HMPV, HCoV, HBoV	Germany	high
111	125	Lee, C.Y.	Delayed respiratory syncytial virus outbreak in 2020 in Taiwan was correlated with two novel RSV-A genotype ON1 variants.	*Influenza Other Respir. Viruses*	2022	Retrospective, observational	RSV	Taiwan	moderate

112	126	Jelley, L.	Spatial and temporal transmission dynamics of respiratory syncytial virus in New Zealand before and after the COVID-19 pandemic.	*Nat. Commun.*	2024	Retrospective, observational	RSV	New Zealand	high
113	127	Pierangeli, A.	Genetic diversity and its impact on disease severity in respiratory syncytial virus subtype-A and -B bronchiolitis before and after pandemic restrictions in Rome.	*J. Infect.*	2023	Prospective, observational	ADV, IFV, RSV, PIV, HRV, HCoV, HBoV	Italy	moderate
114	128	Guo, Y.J.	Epidemiological and genetic characteristics of respiratory syncytial virus infection in children from Hangzhou after the peak of COVID-19.	*J. Clin. Virol.*	2023	Retrospective, observational	RSV	China	moderate
115	129	Cai, W.	Atypical age distribution and high disease severity in children with RSV infections during two irregular epidemic seasons throughout the COVID-19 pandemic, Germany, 2021 to 2023.	*Euro Surveill.*	2024	Prospective, observational	RSV	Germany	high

116	130	Pierangeli, A.	Sequence analysis of respiratory syncytial virus cases reveals a novel subgroup -B strain circulating in north-central Italy after pandemic restrictions.	*J. Clin. Virol.*	2024	Retrospective, observational	RSV	Italy	moderate
117	131	Jiang, M.L.	Changes in endemic patterns of respiratory syncytial virus infection in pediatric patients under the pressure of nonpharmaceutical interventions for COVID-19 in Beijing, China.	*J. Med. Virol.*	2023	Retrospective, observational	RSV	China	moderate
118	132	Chang, D.	The emergence of influenza B as a major respiratory pathogen in the absence of COVID-19 during the 2021-2022 flu season in China.	*Virol. J.*	2023	Retrospective, observational	IFV	China	high
119	133	Sun, Y.	Pathogen characteristics of respiratory infections in the season after the COVID-19 pandemic between August and December 2023:	*Int. J. Infect. Dis.*	2024	Prospective, observational	ADV, IFV, RSV, PIV, HRV, HCoV, SARS-CoV-2	China	moderate

			evidence from direct-to-consumer testing-based surveillance in Guangzhou and Beijing, China.						
120	134	Luštrek, M.	Influenza A, Influenza B, human respiratory syncytial virus and SARSCoV-2 molecular diagnostics and epidemiology in the post COVID-19 era.	*Respir. Res.*	2024	Retrospective, observational	IFV, RSV, SARS-CoV-2	Slovenia	moderate
121	135	Kandeel, A.	Resurgence of influenza and respiratory syncytial virus in Egypt following two years of decline during the COVID-19 pandemic: outpatient clinic survey of infants and children, October 2022.	*B.M.C. Public Health*	2023	Retrospective, observational	IFV, RSV, SARS-CoV-2	Egypt	moderate
122	136	Ouafi, M.	Rapid syndromic testing for respiratory viral infections in children attending the emergency department during COVID-19 pandemic in Lille, France, 2021-2022.	*J. Clin. Virol.*	2022	Retrospective, observational	RSV, HRV, SARS-CoV-2	France	moderate

123	137	Juárez, X.	Children with influenza admitted at a children's hospital in Argentina in the 2019-2022 period: What has changed after the COVID-19 pandemic?	*Arch. Argent. Pediatr.*	2024	Retrospective, observational	IFV, RSV, SARS-CoV-2	Argentina	moderate
124	138	Maison, N.	The rising of old foes: impact of lockdown periods on "non-SARS-CoV-2" viral respiratory and gastrointestinal infections.	*Infection*	2022	Retrospective, observational	ADV, IFV, RSV, HRV,	Germany	moderate
125	139	Kurz, H.	Respiratory Syncytial Virus and Influenza During the COVID-19 Pandemic: A Two-center Experience.	*Pediatr. Infect. Dis. J.*	2024	Retrospective, observational	IFV, RSV	Austria	high
126	140	Olsen, S.J.	Changes in influenza and other respiratory virus activity during the COVID-19 pandemic-United States, 2020-2021.	*Am. J. Transplant.*	2021	Retrospective, observational	ADV, IFV, RSV, PIV, HRV, HCoV, HMPV	USA	moderate
127	141	Zheng, L.	Changes in Influenza Activity and Circulating Subtypes During the COVID-19 Outbreak in China.	*Front. Med.*	2022	Retrospective, observational	IFV	China	high

128	142	Del Riccio, M.	Post-disappearance scenarios: policy implications following the potential disappearance of B/Yamagata lineage influenza viruses.	*Euro Surveill.*	2024	Commentary	-	-	-
129	144	Poole, S.	Physical distancing in schools for SARS-CoV-2 and the resurgence of rhinovirus.	*Lancet Respir. Med.*	2020	Retrospective, observational	HRV	United Kingdom	moderate
130	145	Park, S.	Shifting Patterns of Respiratory Virus Activity Following Social Distancing Measures for Coronavirus Disease 2019 in South Korea.	*J. Infect. Dis.*	2021	Retrospective, observational	ADV, IFV, PIV, RSV, HCoV, HRV, HBoV, HMPV	South Korea	moderate
131	146	Thongpan, I.	Upsurge of human rhinovirus infection followed by a delayed seasonal respiratory syncytial virus infection in Thai children during the coronavirus pandemic.	*Influenza Other Respir. Viruses*	2021	Retrospective, observational	RSV, HRV	Thailand	moderate
132	147	Zhang, R.X.	Surges of hospital-based rhinovirus infection during the 2020 coronavirus disease-19 (COVID-19)	*World J. Pediatr.*	2021	Retrospective, observational	ADV, IFV, HRV, RSV,	China	moderate

			pandemic in Beijing, China.						
133	148	Giannattasio, A.	Rhinovirus in pediatric respiratory infections: More than a simple cold.	*J. Med. Virol.*	2024	Retrospective, observational	ADV, IFV, RSV, PIV, HRV, HCoV, HMPV	Italy	moderate
134	149	Presti, S.	Comparative Analysis of Pediatric Hospitalizations during Two Consecutive Influenza and Respiratory Virus Seasons Post-Pandemic.	*Viruses*	2023	Retrospective, observational	ADV, IFV, RSV, PIV, HRV, HCoV, HMPV, HBoV	Italy	high
135	150	Maison, N.	Old foes following news ways?-Pandemic-related changes in the epidemiology of viral respiratory tract infections.	*Infection*	2024	Retrospective, observational	ADV, IFV, RSV, PIV, HRV, HCoV, HMPV, SARS-CoV-2	Germany	moderate
136	151	Gil, E.	Increasing rhinovirus prevalence in paediatric intensive care patients since the SARS-CoV2 pandemic.	*J. Clin. Virol.*	2023	Retrospective, observational	HRV	United Kingdom	high
137	152	Yoon, Y.	Stepwise School Opening and an Impact on the Epidemiology of COVID-19 in the Children.	*J. Korean Med. Sci.*	2020	Retrospective, observational	SARS-CoV-2	South Korea	moderate

138	153	Cho, H.J.	Epidemiology of Respiratory Viruses in Korean Children Before and After the COVID-19 Pandemic: A Prospective Study From National Surveillance System.	*J. Korean Med. Sci.*	2024	Prospective, observational	ADV, IFV, RSV, PIV, HRV, HCoV, HMPV, HBoV,	South Korea	moderate
139	154	Kim, E.Y.	Children with COVID-19 after Reopening of Schools, South Korea. *Pediatr. Infect.*	*Vaccine*	2020	Retrospective, observational	SARS-CoV-2	South Korea	moderate
140	155	Haapanen, M.	The impact of the lockdown and the re-opening of schools and day cares on the epidemiology of SARS-CoV-2 and other respiratory infections in children-A nationwide register study in Finland.	*EClinical Medicine*	2021	Retrospective, observational	ADV, RSV, IFV, PIV, HRV, SARS-CoV-2	Finland	high
141	156	Foley, D.A.	An unusual resurgence of human metapneumovirus in Western Australia following the reduction of non-pharmaceutical interventions to prevent	*Viruses*	2022	Retrospective, observational	HMPV	Australia	high

			SARS-CoV-2transmission						
142	157	Foley, D.A.	A surge in human metapneumovirus paediatric respiratory admissions in Western Australia following the reduction of SARS-CoV-2 nonpharmaceutical interventions.	*J. Paediatr. Child Health*	2023	Retrospective, observational	HMPV	Australia	moderate
143	158	Pelletier, J.H.	Trends in US Pediatric Hospital Admissions in 2020 Compared With the Decade Before the COVID-19 Pandemic.	*J.A.M.A. Netw. Open*	2021	Retrospective, observational	-	USA	moderate
144	159	Sachs, N.	The Effect of the COVID-19 Pandemic on Pediatric Respiratory Hospitalizations.	*Isr. Med. Assoc. J.*	2023	Retrospective, observational	IFV, RSV	Israel	moderate
145	160	Hartnett, K.P.	Impact of the COVID-19 Pandemic on Emergency Department Visits - United States, January 1, 2019-May 30, 2020.	*M.M.W.R. Morb. Mortal Wkly Rep.*	2020	Retrospective, observational	-	USA	moderate
146	161	Liu, P.	Impact of COVID-19 pandemic on the prevalence of respiratory viruses in	*Virol. J.*	2021	Retrospective, observational	ADV, RSV, PIV, IFV, HMPV, HRV	China	high

			children with lower respiratory tract infections in China.						
147	162	van Summeren, J.	Low levels of respiratory syncytial virus activity in Europe during the 2020/21 season: what can we expect in the coming summer and autumn/winter?	*Euro Surveill.*	2021	Retrospective, observational	RSV	-	high
148	163	Amaddeo, A.	Social distancing measures for COVID-19 are changing winter season.	*Arch. Dis. Child.*	2021	Retrospective, observational	ADV, IFV, RSV, PIV, HRV, HCoV, HMPV, HBoV, SARS-CoV-2	France	moderate

ADV; Adenovirus; IFV: Influenza Virus; RSV; Respiratory Syncitial Virus; PIV: Parainfluenza Virus; HMPV: Human Metapneumovirus; SARS-CoV-2: Severe Acute Respiratory Syndrome Coronavirus 2; HCoV: non-SARS-CoV-2 Human Coronaviruses, HBoV: Human Bocavirus; HRV: Human Rhinovirus; PEV: Paraechovirus; MERS: Middle East Respiratory Syndrome Virus.

Supplementary Table 2. Articles initially included during the literature screening but then excluded from the final selection.

No.	First Author	Title	Journal	Year	Reason for exclusion	Country
1	Lebreiro, G.P.	Respiratory syncytial virus infection in children during SARS-CoV-2 pandemic at a referral center in Rio de Janeiro, Brazil.	*J. Bras. Pneumol.*	2024	Focus limited to the comparison between SARS-CoV-2 and RSV infection severity	Brazil
2	Roland, D.	Features of the transposed seasonality of the 2021 RSV epidemic in the UK and Ireland: analysis of the first 10 000 patients.	*Arch. Dis. Child.*	2022	Free full text unavailable	United Kingdom, Ireland
3	Nunziata, F.	Clinical Presentation and Severity of SARS-CoV-2 Infection Compared to Respiratory Syncytial Virus and Other Viral Respiratory Infections in Children Less than Two Years of Age.	*Viruses*	2023	Focus limited to the comparison between SARS-CoV-2 and RSV	Italy
4	Li, W.	Genetic diversity and epidemiology of human rhinovirus among children with severe acute respiratory tract infection in Guangzhou, China.	*Virol. J.*	2021	Focus limited to HRV genotypes	China
5	Wang, Q.	High Diversity in Genotypes of Human Rhinovirus Contributes to High Prevalence in Beijing, 2018-2022: A Retrospective Multiple-Center Epidemiological Study.	*Biomed. Environ. Sci.*	2024	Focus limited to HRV genotypes	China
6	Parums, D.V.	Editorial: Outbreaks of Post-Pandemic Childhood Pneumonia	*Med. Sci. Monit.*	2023	Focus on bacterial infections	-

		and the Re-Emergence of Endemic Respiratory Infections.				
7	Sitthikarnkha, P.	Epidemiology of acute lower respiratory tract infection hospitalizations in Thai children: A 5-year national data analysis.	*Influenza Other Respir. Viruses*	2022	No data related to the COVID-19 pandemic	Thailand
